# Shared mechanisms and crosstalk of COVID-19 and osteoporosis via vitamin D

**DOI:** 10.1038/s41598-022-23143-7

**Published:** 2022-10-28

**Authors:** Fei Liu, Chao Song, Weiye Cai, Jingwen Chen, Kang Cheng, Daru Guo, Dayue Darrel Duan, Zongchao Liu

**Affiliations:** 1 Department of Orthopedics, The Affiliated Hospital of Traditional Chinese Medicine of Southwest Medical University, Luzhou, 646000 Sichuan China; 2 Center for Phenomics of Traditional Chinese Medicine, and the Affiliated Hospital of Traditional Chinese Medicine of Southwest Medical University, Luzhou, 646000 Sichuan China

**Keywords:** Diseases, Immunological disorders, Infectious diseases, Drug development, Epidemiology

## Abstract

Recently accumulated evidence implicates a close association of vitamin D (VitD) insufficiency to the incidence and clinical manifestations of the COVID-19 caused by severe acute respiratory syndrome coronavirus-2 (SARS-COV-2). Populations with insufficient VitD including patients with osteoporosis are more susceptible to SARS-COV-2 infection and patients with COVID-19 worsened or developed osteoporosis. It is currently unknown, however, whether osteoporosis and COVID-19 are linked by VitD insufficiency. In this study, 42 common targets for VitD on both COVID-19 and osteoporosis were identified among a total of 243 VitD targets. Further bioinformatic analysis revealed 8 core targets (EGFR, AR, ESR1, MAPK8, MDM2, EZH2, ERBB2 and MAPT) in the VitD-COVID-19-osteoporosis network. These targets are involved in the ErbB and MAPK signaling pathways critical for lung fibrosis, bone structural integrity, and cytokines through a crosstalk between COVID-19 and osteoporosis via the VitD-mediated conventional immune and osteoimmune mechanisms. Molecular docking confirmed that VitD binds tightly to the predicted targets. These findings support that VitD may target common signaling pathways in the integrated network of lung fibrosis and bone structural integrity as well as the immune systems. Therefore, VitD may serve as a preventive and therapeutic agent for both COVID-19 and osteoporosis.

## Introduction

Coronavirus disease 2019 (COVID-19) is an infectious disease caused by the novel severe acute respiratory syndrome coronavirus-2 (SARS–COV-2)^1,2^. Susceptibility of human to SARS-COV-2 has caused a global COVID-19 pandemic since the beginning of 2020, which spreads rapidly and has a relatively high fatality rate^3^. As of February 10, 2022, there have been 430,257,564 confirmed cases of COVID-19, including 5,922,049 deaths (infection fatality ratio (IFR) of 5,922,049/430,257,564 × 100 = 1.376%) from over 223 countries or regions reported to the World Health Organization (WHO) (https://covid19.who.int/). So far, no effective drug has been found for COVID-19 and the clinical treatment is mainly to relieve symptoms and maintain basic vital signs^4^. Although various vaccines against SARS–COV-2 have been widely used and have been shown to be effective to improve human resistance to COVID-19, the duration of vaccine-induced immunity remains largely unknown and the effectiveness of the vaccines as a preventive measure has been seriously challenged by the extremely rapid rate of virus mutations^5^. Interestingly, it was reported that low plasma 25(OH)-vitamin D (VitD) level or hypovitaminosis D is an independent risk factor for COVID-19 incidence and hospitalization^6^. Hypovitaminosis D was associated with a higher prevalence in patients with severe infection and it was predictive of hospitalization and mortality^7^. On the other hand, supplement of VitD in the treatment regimen for COVID-19 patients achieved certain therapeutic effects^8^ and reduced inflammatory markers^9^. The immunomodulatory function of VitD was effective in preventing multiple organ failure, cardiovascular complications and other side effects caused by cytokine storm in COVID-19 patients^10^. These evidence indicate a close association of VitD to the incidence and clinical manifestations of the COVID-19. Recently, it was found that osteoporosis patients were more susceptible to SARS-COV-2 infection and had worse osteoporosis manifestations after suffering from COVID-19 while some COVID-19 patients developed osteoporosis as a complication^11^. It is well known that VitD insufficiency is the key causative factor for osteoporosis and VitD is one of the major therapeutic agents in the treatment of osteoporosis^12^. It is currently unknown, however, whether and how VitD insufficiency links osteoporosis with COVID-19. In this study, multiple approaches, including network pharmacology, bioinformatics, and molecular docking were used to identify and characterize the targets of VitD on COVID-19 and osteoporosis related diseases and to explore the molecular mechanism for the potential therapeutic effects of VitD in the treatment of both COVID-19 and osteoporosis (see Supplementary Fig. 0).

## Materials and methods

### Acquisition of VitD targets

The relevant chemical information and several potential targets of VitD were searched through the PubChem database (https://pubchem.ncbi.nlm.nih.gov/)^13^, and the chemical structure of VitD was downloaded using PubChem and saved in pdbqt file. The pdbqt file of VitD was input into Pharmmapper (http://lilab-ecust.cn/pharmmapper/)^14^ to predict its potential targets of VitD. With "Vitamin D" as the key word, collected potential targets of VitD in Drugbank (https://go.drugbank.com/)^15^ and Swiss target prediction (https://www.expasy.org/resources/swisstargetprediction)^16^. The targets of VitD were imported into UniProt^17^ (http://www.uniprot.org/) to obtain the official gene symbol. Finally, those targets were combined and deduplicated to obtain the set of VitD potential targets.

### Identified of osteoporosis and COVID-19 targets

COVID-19 disease genes were searched and collected using the NCBI database^18^ (http://omim.org/), TTD^19^ (http://db.idrblab.net/ttd/) and Genecards (http://www.genecards.org)^20^. The search results of those database were merged and deduplicated to obtain COVID-19 gene set. Osteoporosis disease genes were searched and collected using the NCBI database^18^ (http://omim.org/), Disgenet^21^ (https://www.disgenet.org/home/) and Genecards^22^ (http://www.genecards.org). The search results of those database were merged and deduplicated to obtain osteoporosis gene set.

### Screening of common drug-disease targets

The VitD targets and osteoporosis and COVID-19 genes are shown in Supplementary Table S1–S10. The potential target set of VitD was compared with those of osteoporosis and COVID-19, and the overlap among the three was used as the targets of VitD for both osteoporosis and COVID-19.

### Construction and analysis of the "drug-target-disease" (DTD) network

Cytoscape 3.7.2 software^22^ was used to construct the network diagram of "drug-target-disease" relationship. The common drug-disease targets were input to String database (https://string-db.org/), a database for searching protein interactions, including both direct physical interactions between proteins and indirect functional correlations^23^, to collect the protein–protein interaction (PPI) data and construct PPI network diagram. The PPI data was analyzed through the "Network Analyzer" function under "Tool", and the node degree centrality were used to reflect the importance of the node. The connection between targets indicates the interaction relationship of DAVID^24^. The relevant feature data were recorded for subsequent analysis. After the completion of the DTD network construction, the analysis files were downloaded and imported into Cytoscape 3.7.2 software^22^, and the core targets of VitD in the treatment of osteoporosis and COVID-19 were obtained by screening according to set the value of twice the nodal degree (degree ≥ 10.2)^25^.

### Gene ontology (GO) and KEGG pathway enrichment analyses of core targets

In order to further understand the functions of core target genes and the main pathways of VitD in the treatment of osteoporosis and COVID-19, R language was used to acquire Gene ontology (GO) enrichment analysis and KEGG pathway enrichment analysis of core targets^26^, and the species were selected as “Homo sapiens”. To further accurately localize the signaling pathways involved in the pathogenic mechanisms of COVID-19 and osteoporosis and their interaction with Vit D, the core targets obtained were imported to the two signaling pathway databases supplement to the KEGG and GO signaling pathway databases, WikiPathways^27^ and Rectome^28^, respectively, and a list of signaling pathways where the targets were located was obtained separately. The signaling pathways obtained from WikiPathways were sorted from highest to lowest background gene counts, and those obtained from Rectome were sorted from highest to lowest *p* values.

### Molecular docking of predicted targets

1. Ligand processing: The 3D structure of VitD in mol2 format was obtained from Pubchem database, the small ligand molecule was hydrogenated, charged, ligand roots were detected, rotatable bonds were searched and defined using AutodockTools 1.5.6^29^, and later saved as pdbqt files.
2. Receptor Processing: the core three-dimensional (3D) structure of the target proteins from RCSB protein database (www.rcsb.org/)^30^ was download as a docking protein. The 3D structure was opened by adding all hydrogen atoms in AutodockTools 1.5.6, Gasteiger charge was calculated, the nonpolar hydrogen was combined, and a receptor was defined and saved as a pdbqt file.
3. Docking Parameter setting: the coordinates and box size of Vina molecule docking was determined, the parameter exhaustiveness was set to 15, and other parameters were taken as default values.
4. Operation and Output: Autodockvina 1.1.2 was used for semi-flexible docking, and the conformation with the best affinity was selected as the final docking conformation.

## Results

### Identification of common targets for COVID-19, Osteoporosis, and VitD

**Figure 1:**
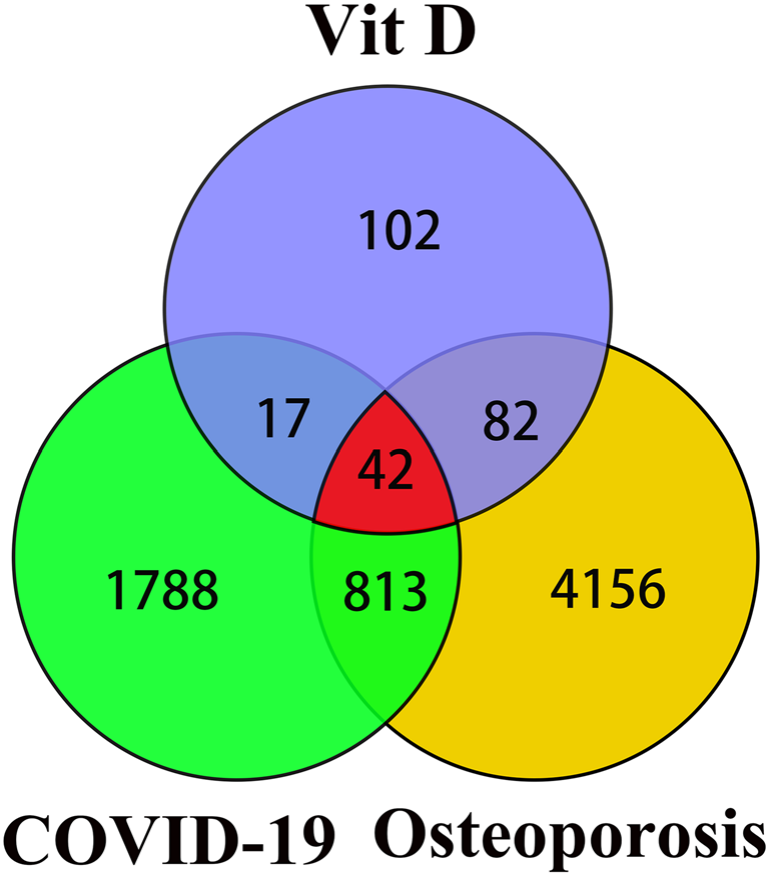
Venny diagram of the association of VitD targets with osteoporosis and COVID-19 targets. A total of 243 VitD targets, 2660 COVID-19 targets, and 5093 osteoporosis targets were identified. There exist 855 common targets between COVID-19 and osteoporosis, 59 common targets between VitD and COVID-19, 124 common targets between VitD and osteoporosis, and 42 common targets among VitD, COVID-19 and osteoporosis.

For the COVID-19 targets, 106 related targets were acquired in TTD, 244 in NCBI and 2571 in GeneCrds; after removing duplicates, 2660 targets were identified. For the osteoporosis targets, 1098 related targets were acquired in DisGenet, 639 in NCBI, and 4576 in GeneCards; after removing duplicates, 5093 targets were identified. A total of 243 VitD potential targets were acquired after deduplication from our database. COVID-19 and osteoporosis shared 855 common targets; VitD and COVID-19 shared 59 common targets; and VitD and osteoporosis shared 124 common targets; and 42 common targets of VitD, COVID-19, and osteoporosis were identified by intersecting the acquired targets(Fig. 1). Among the 42 common targets, all could interact with each other. Hence, these 42 targets were used to construct the VitD-COVID-19-osteoporosis PPI network (the above basic data are from Supplementary Table S11–S14).

### The "drug-target-disease" (DTD) network and core targets

**Figure 2:**
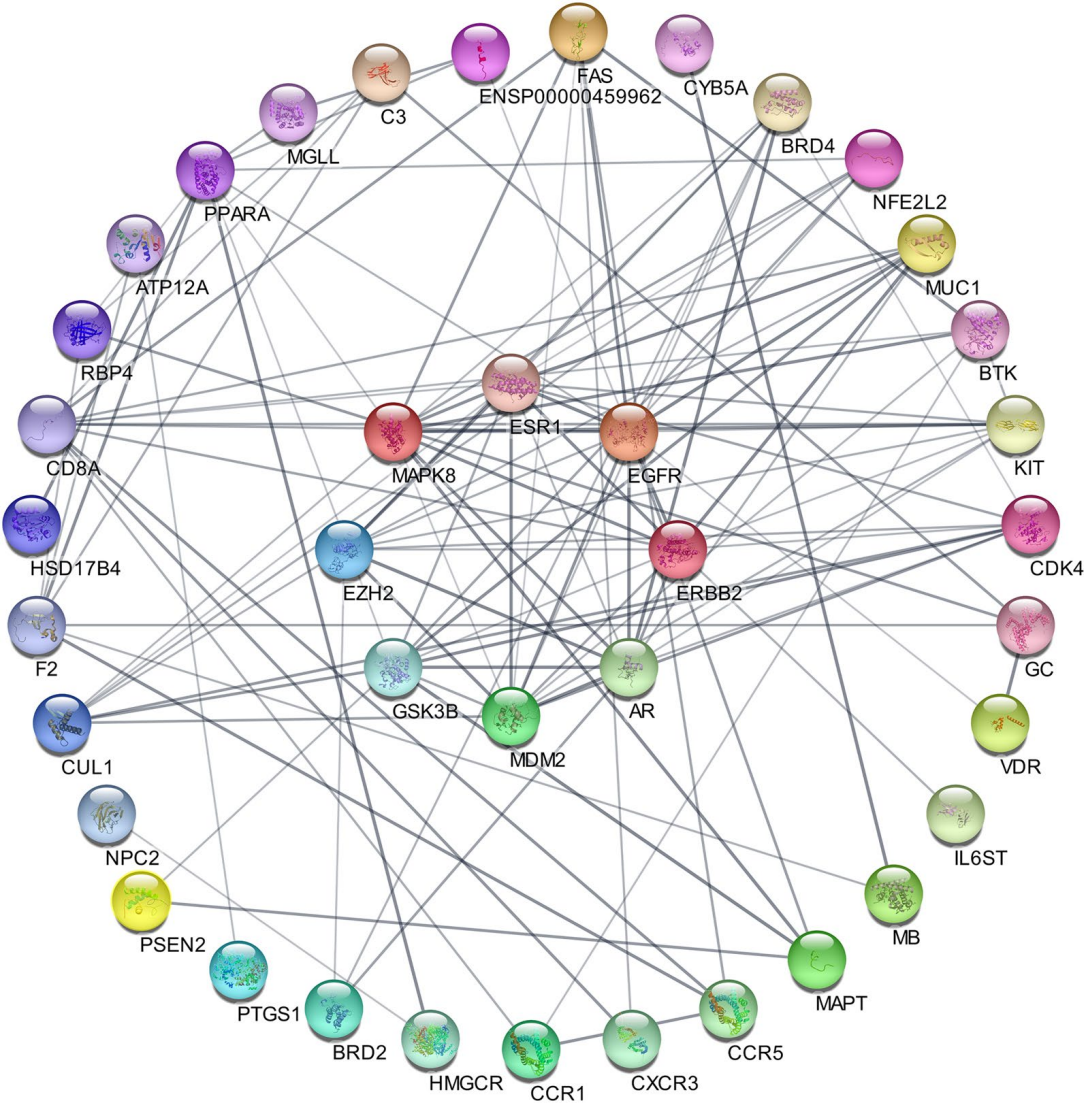
Protein–protein-interaction (PPI) map of "Drug-Targets-Disease”. Network nodes represent proteins, i.e. each node represents all proteins produced by a single protein-coding locus. The edges represent protein–protein associations, where associations are meant to be specific and meaningful, i.e. proteins together contribute to a common function. Network Statistics: Nodes: 42, Edges: 117, Average Node Degree: 5.57, Local Clustering Coefficient: 0.53, Expected Edges: 56, PPI Enrichment p-Value: 7.72e-13.

**Figure 3:**
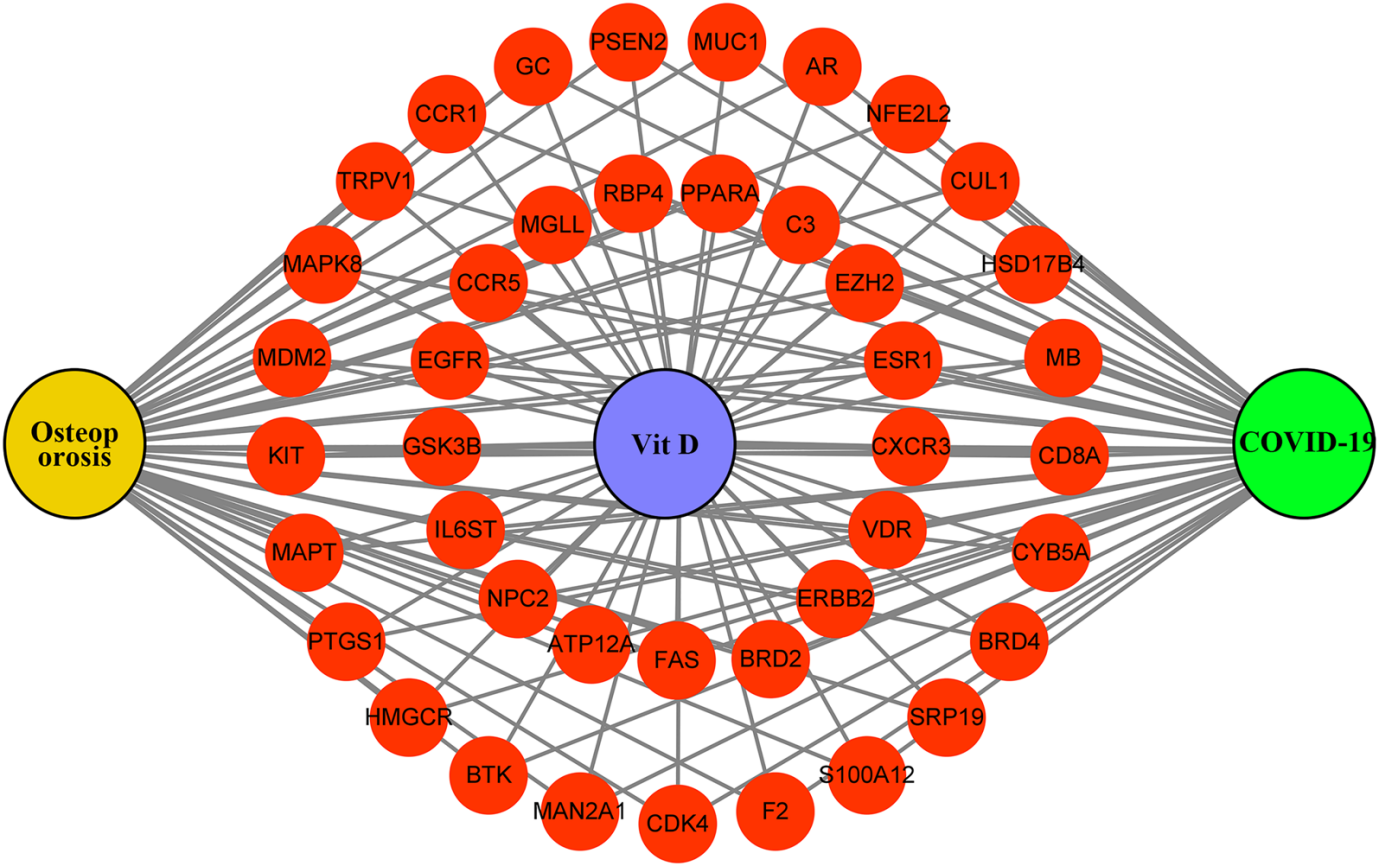
Drug-Targets-Disease Interaction Network. Graphs were constructed to link VitD to osteoporosis and COVID-19 targets. VitD can act on osteoporosis and COVID-19 through the 42 targets shown.

To further elucidate the mechanisms for VitD in the treatment of COVID-19 and osteoporosis, the 42 common targets of VitD, osteoporosis, and COVID-19 was imported to the STRING online service platform, and the DTD network data were visualized and analyzed via the Analysis network tool in Cytoscape 3.7.2 software. A total of 42 nodes and 108 edges and 5.1 the average node degree were included in the PPI network (Fig. 2). The targets that did not intersect with COVID-19 and osteoporosis were removed, and the interaction diagram of "drug-target-disease" was made (Fig. 3).

**Figure 4:**
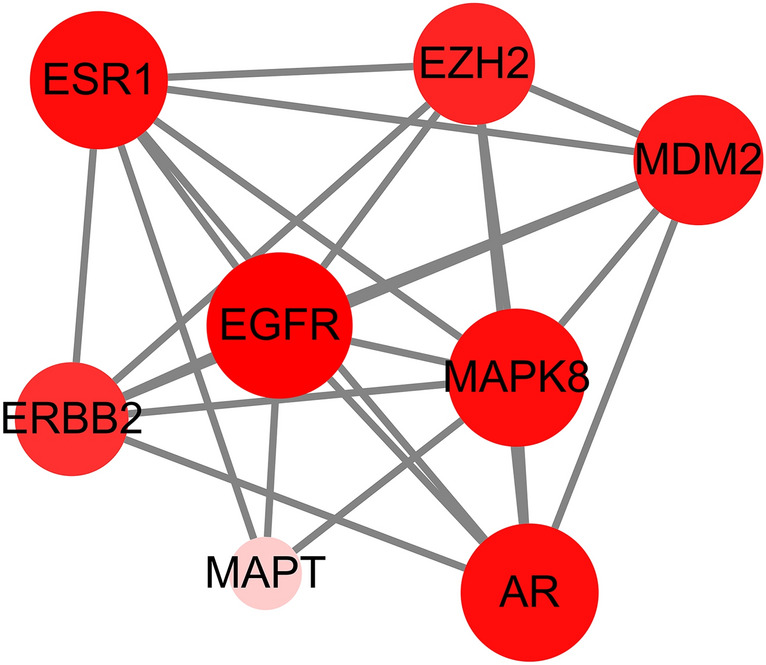
Core targets. This figure shows the core target map obtained based on 42 common targets constructed by PPI, based on topological heterogeneity analysis and setting twofold average node degree. Among them, EGFR and MAPK targets have the most interactions with other targets among the core targets, and thus they play a crucial role in the treatment of diseases.

When the core targets were screened by the topological features^31^ and twice the average node degree as the screening criteria (degree ≥ 10.2), epidermal growth factor receptor (EGFR), AR, ESR1, MAPK8, MDM2, EZH2, ERBB2, and MAPT were identified as the core targets of VitD, osteoporosis, and COVID-19 (Supplementary Table S15). A new DTD network including the 8 core targets was acquired after the screening (Fig. 4).

### Shared signaling pathways and crosstalk between COVID-19 and osteoporosis via VitD

**Figure 5:**
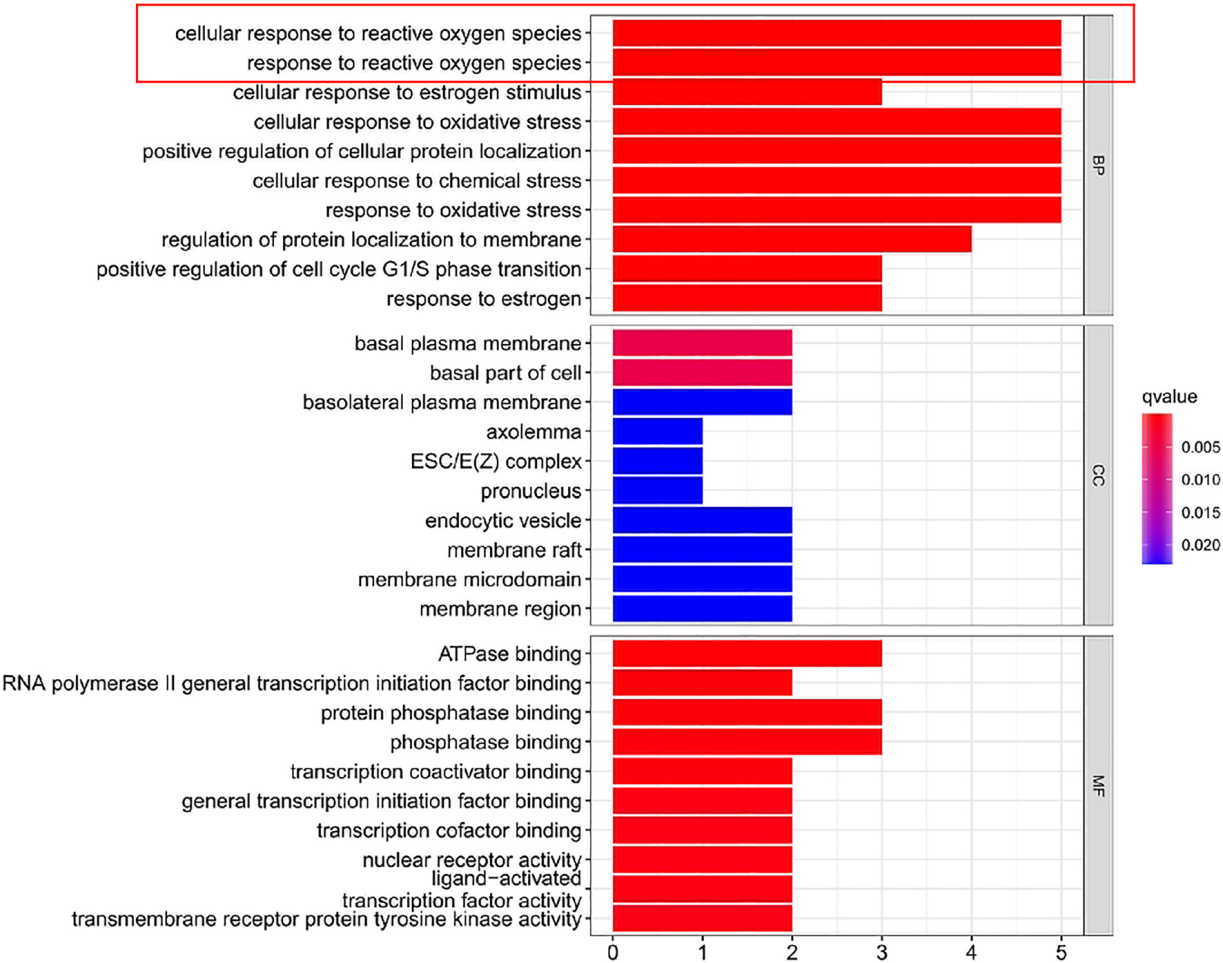
Advanced histogram of GO biological process analysis. In this study, 724 GO terms were identified, including 632 biological processes (BP), 26 cellular components (CC) and 66 molecular functions (MF). The top 10 most important terms for BP, CC and MF are clearly shown by the bar chart.

**Figure 6:**
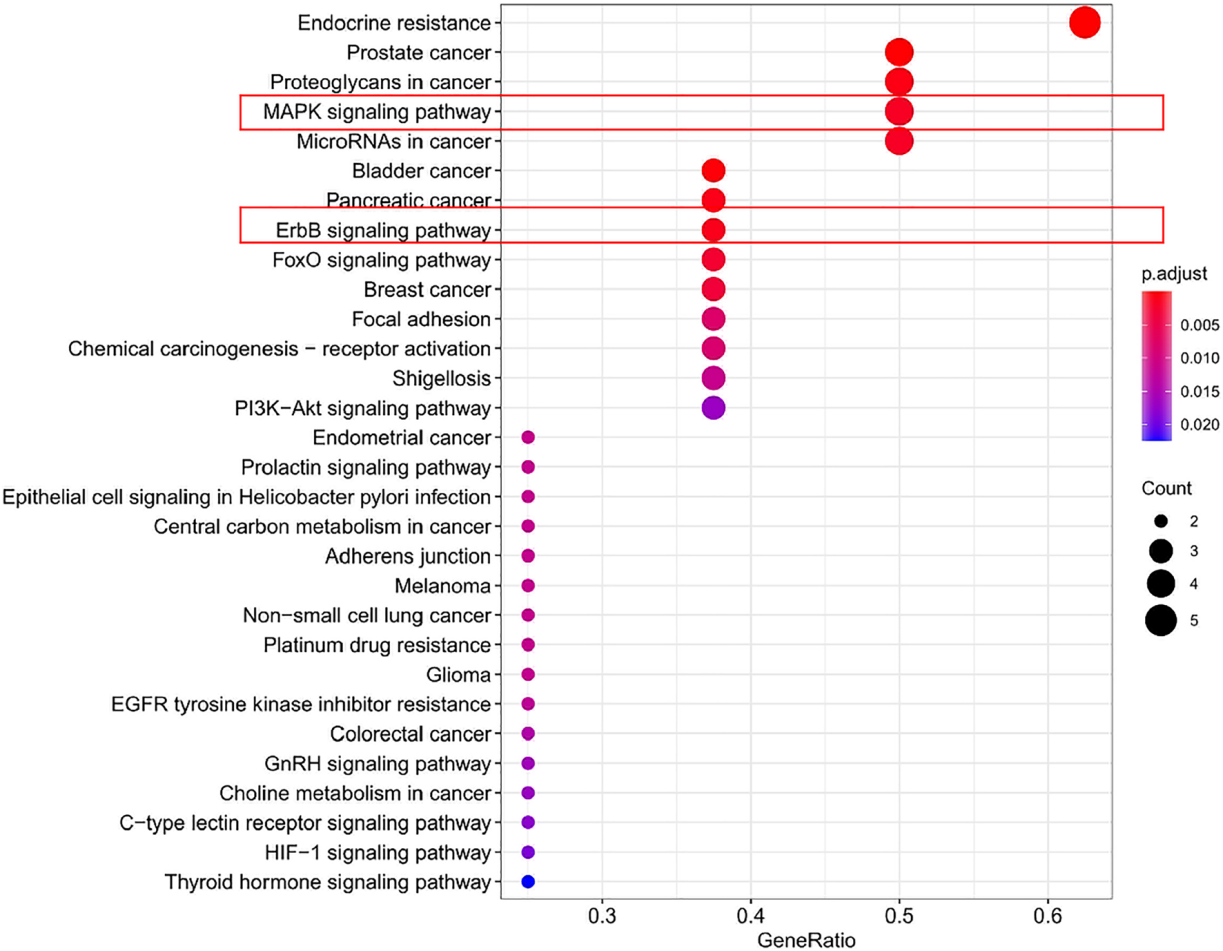
Advanced bubble diagram of KEGG pathway enrichment analysis. By KEGG pathway enrichment analysis, 34 pathways were obtained, and the top 10 important pathways were shown by bubbles through screening.

GO and KEGG enrichment analyses of the 8 common core targets of VitD, osteoporosis, and COVID-19 were performed by R language (Supplementary Table S16–17). In this study, 724 GO entries were found, including 632 for biological process (BP), 26 for cellular components (CC) and 66 for molecular function (MF). The top 10 most important terms of BP, CC, and MF are shown in Fig. 5**.** BP terms mainly included cellular responses to reactive oxygen species (ROS), chemical stress, protein localization to the membrane, cell cycle G1/phase transition, estrogen stimulus, and other biological processes. CC terms were mainly enriched in membrane microdomain, membrane raft, endocytic vesicle, Pronucleus, ESC/E(Z) complex, Axolemma, basolateral plasma membrane, basal part of cell, basal plasma membrane, membrane region. MF terms mainly involved ATPase binding, transmembrane receptor protein tyrosine kinase activity, ligand-activated, transcription factor activity, nuclear receptor activity, transcription cofactor binding, general transcription initiation factor binding, transcription coactivator binding, phosphatase binding, protein phosphatase binding, RNA polymerase II general transcription initiation factor binding (Table 1)**.** By the KEGG pathway enrichment analysis, 34 pathways were obtained, and the top 10 pathways with an important role were acquired by screening (Fig. 6). Potential targets for VitD anti-treatment osteoporosis and COVID-19 were enriched mainly in ErbB signaling pathway, MAPK signaling pathway, FoxO signaling pathway, endocrine resistance, and several pathways related to cancers (prostate cancer, bladder cancer, proteoglycans in cancer, pancreatic cancer, microRNAs in cancer, and breast cancer) (Table 2).

**Table 1:** GO enrichment analysis of core targets.

Type	Numbering	Pathway of action	qvalue	Number of genes
Bioprocess	GO:0034614	Cellular response to reactive oxygen species	9.43E−07	5
Bioprocess	GO:0000302	Response to reactive oxygen species	2.40E−06	5
Bioprocess	GO:0071391	Cellular response to estrogen stimulus	2.86E−06	3
Bioprocess	GO:0034599	Cellular response to oxidative stress	4.79E−06	5
Bioprocess	GO:1903829	Positive regulation of cellular protein localization	5.90E−06	5
Bioprocess	GO:0062197	Cellular response to chemical stress	6.73E−06	5
Bioprocess	GO:0006979	Response to oxidative stress	1.91E−05	5
Bioprocess	GO:1905475	Regulation of protein localization to membrane	2.88E−05	4
Bioprocess	GO:1902808	Positive regulation of cell cycle G1/S phase transition	5.78E−05	3
Bioprocess	GO:0043627	Response to estrogen	0.000102393	3

**Table 2:** KEGG enrichment analysis of core targets.

Term	Signal path	p.adjust	Number of genes
hsa01522	Endocrine resistance	1.48E−06	5
hsa05215	Prostate cancer	7.55E−05	4
hsa05205	Proteoglycans in cancer	0.00074556	4
hsa04010	MAPK signaling pathway	0.00175415	4
hsa05206	MicroRNAs in cancer	0.001886987	4
hsa05219	Bladder cancer	0.00025525	3
hsa05212	Pancreatic cancer	0.000993652	3
hsa04012	ErbB signaling pathway	0.00115851	3
hsa04068	FoxO signaling pathway	0.002802206	3
hsa05224	Breast cancer	0.003545864	3

**Figure 7:**
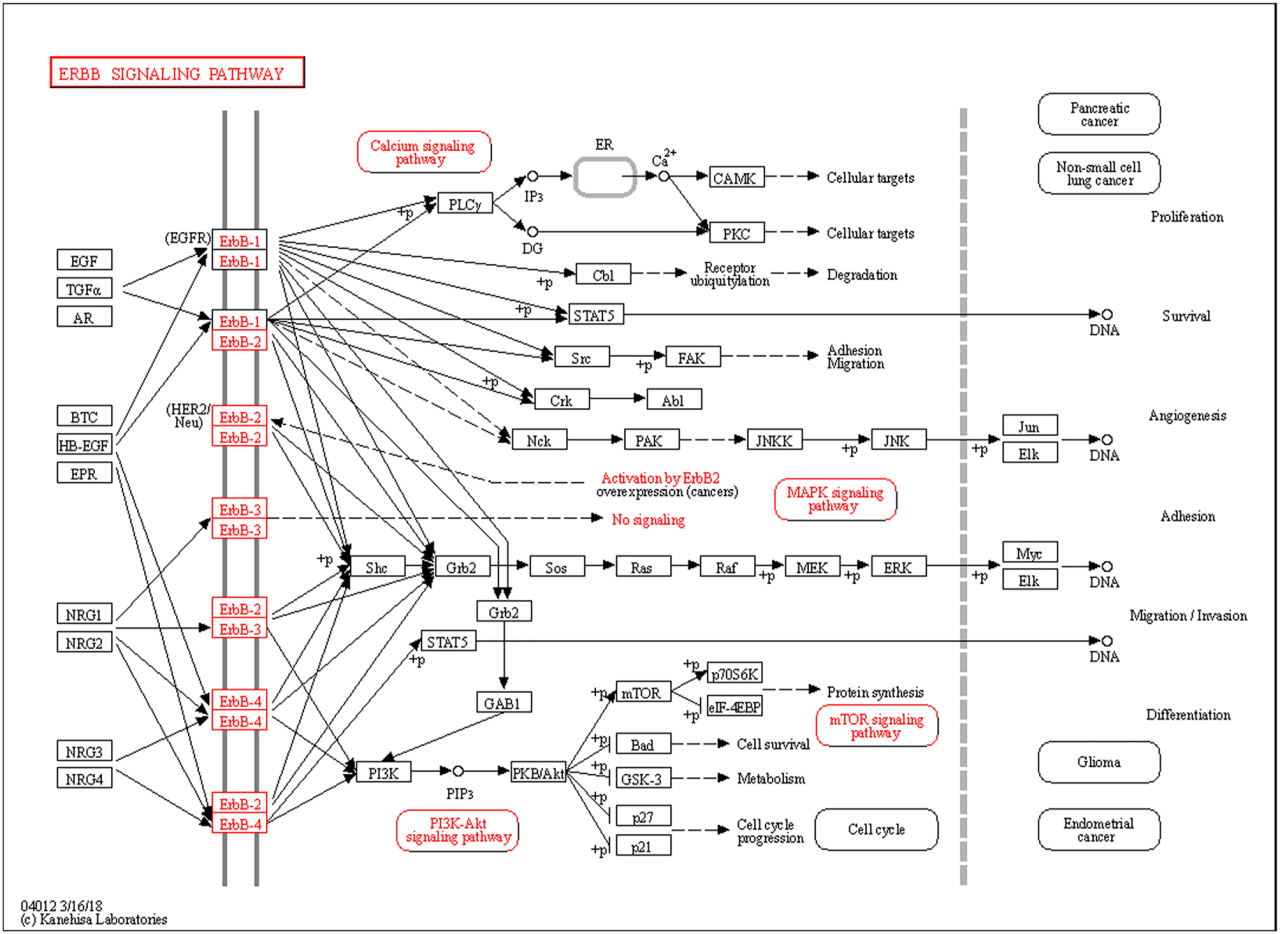
ERBB signaling pathway. The mechanisms of action of the ERBB signaling pathway that has been identified so far.

**Figure 8:**
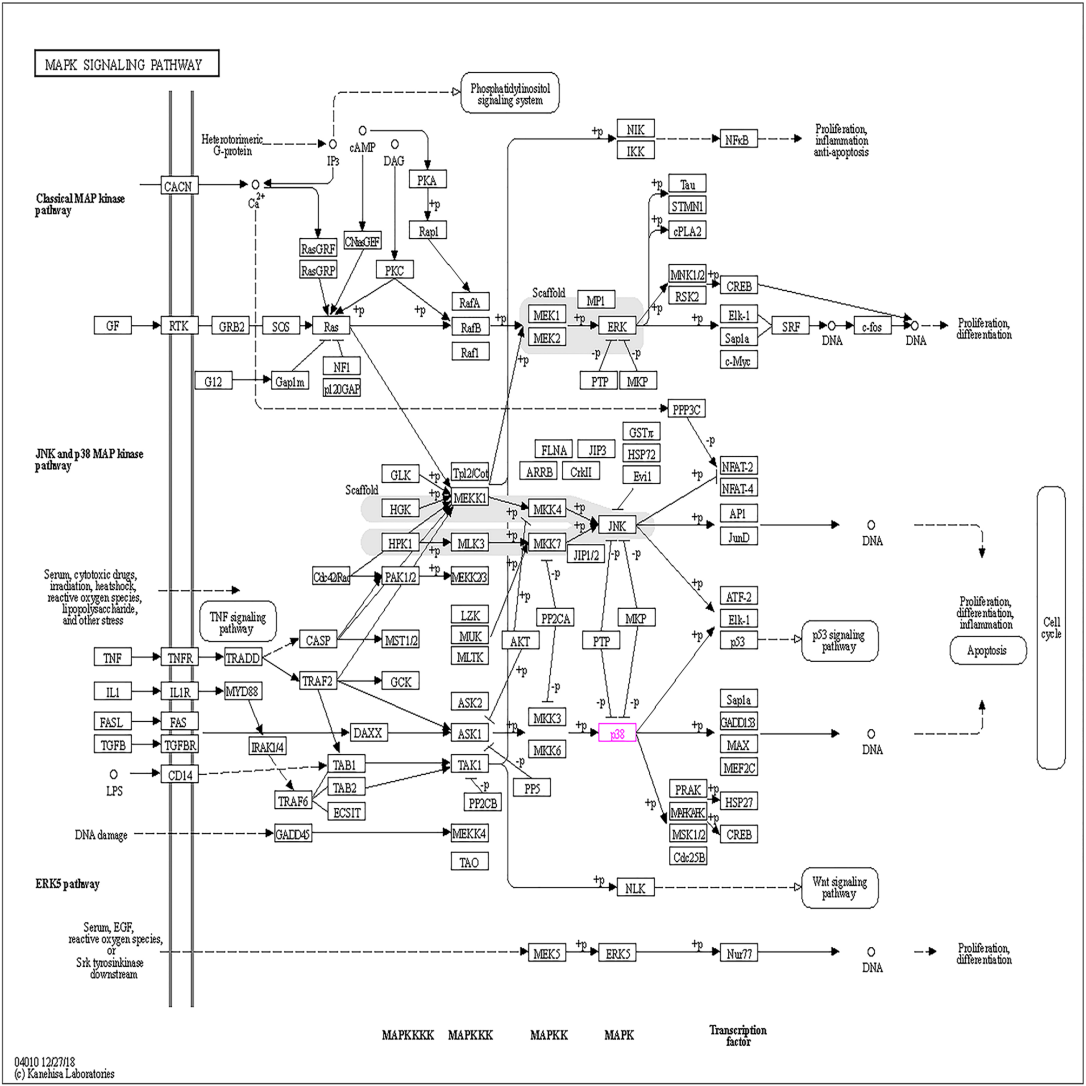
MAPK signaling pathway. The mechanism of action of the MAPK signaling pathway that has been identified so far.

**Figure 9:**
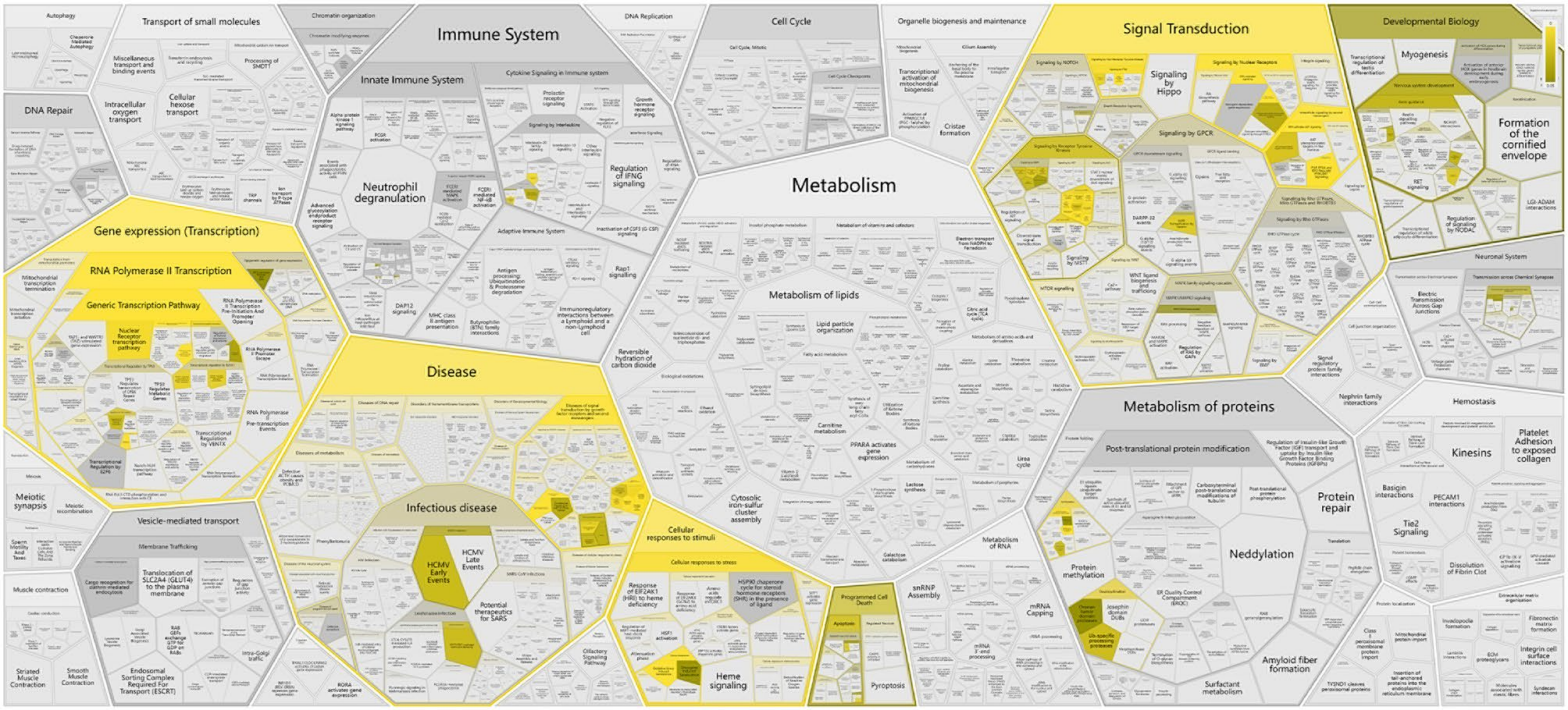
Rectome analysis. The diagrams show the human systems and locations of cellular responses involved in the core targets' actions, etc. The eight core targets focus on the immune system, the innate immune system, cytokine signaling in the immune system, signaling in the GPCR, vesicle-mediated transport, and other signaling pathways. Among these, the mechanisms involved in the immune system make it a priority for us to understand and study in depth.

To further accurately localize the signaling pathways involved in the pathogenic mechanisms of COVID-19 and osteoporosis and their interaction with Vit D, the two signaling pathway databases supplement to the KEGG and GO signaling pathway database, WikiPathways and Rectome, respectively, were used. From the WikiPathways analysis, ERBB signaling pathway (Fig. 7) and mitogen-activated protein kinase (MAPK) signaling pathway (Fig. 8) and related targets, including ErbB, ERBB2, EGFR, mitogen-activated protein kinase 8 (MAPK8) were revealed (Table 3), suggesting the importance of the targets of EGFR and MAPK8 ERBB and MAPK signaling pathways for VitD in its actions on COVID-19 and osteoporosis. From the GO and Rectome analysis(Table 4), the 8 common core targets involved in the innate immune system, cytokine signaling, GPCR signaling, vesicle-mediated transport, immune system and other signaling pathways were revealed (Supplementary Table S18–19), suggesting that the crosstalk between COVID-19 and osteoporosis by VitD may be through the regulation of the cytokine signaling and immune system in COVID-19 patients to improve pulmonary fibrosis and activate osteoimmune mechanisms to regulate the structural integrity of bone (Fig. 9).

**Table 3:** WikiPathways analysis of core targets.

Term ID	Term description	Background gene count	Matching proteins
WP382	MAPK signaling pathway	245	EGFR, MAPT, MAPK8
WP306	Focal adhesion	196	ERBB2, EGFR, MAPK8
WP437	EGF/EGFR signaling pathway	162	ERBB2, EGFR, MAPK8
WP4262	Breast cancer pathway	153	ERBB2, EGFR, ESR1
WP1984	Integrated breast cancer pathway	151	MDM2, EGFR, AR, ESR1
WP4673	Male infertility	141	MDM2, AR, ESR1
WP3931	Embryonic stem cell pluripotency pathways	116	MDM2, EGFR
WP4659	Gastrin signaling pathway	114	EGFR, MAPK8
WP710	DNA damage response (only ATM dependent)	110	MDM2, ERBB2, MAPK8
WP673	ErbB signaling pathway	90	MDM2, ERBB2, EGFR, MAPK8
WP138	Androgen receptor signaling pathway	88	MDM2, EGFR, AR
WP4263	Pancreatic adenocarcinoma pathway	87	ERBB2, EGFR, MAPK8
WP4806	EGFR tyrosine kinase inhibitor resistance	83	ERBB2, EGFR
WP2261	Glioblastoma signaling pathways	82	MDM2, ERBB2, EGFR
WP4538	Regulatory circuits of the STAT3 signaling pathway	78	EGFR, MAPK8
WP2037	Prolactin signaling pathway	76	ERBB2, MAPK8
WP2034	Leptin signaling pathway	75	ERBB2, MAPK8, ESR1
WP4255	Non-small cell lung cancer	72	ERBB2, EGFR
WP4674	Head and neck squamous cell carcinoma	72	ERBB2, EGFR
WP3303	RAC1/PAK1/p38/MMP2 pathway	67	ERBB2, EGFR, MAPK8

**Table 4:** Rectome analysis of core targets.

Pathway identifier	Pathway name	Entities pValue	Entities FDR	Submitted Entities Found
R-HSA-168256	Immune system	0.96433308	0.96433308	MAPK8
R-HSA-168249	Innate immune system	0.791998363	0.791998363	MAPK8
R-HSA-1280215	Cytokine signaling in immune system	0.720199279	0.720199279	MAPK8
R-HSA-372790	Signaling by GPCR	0.632280261	0.632280261	EGFR
R-HSA-5653656	Vesicle-mediated transport	0.615243857	0.615243857	EGFR
R-HSA-388396	GPCR downstream signalling	0.596034093	0.596034093	EGFR
R-HSA-1640170	Cell Cycle	0.570376168	0.570376168	MDM2
R-HSA-9716542	Signaling by Rho GTPases, Miro GTPases and RHOBTB3	0.56578318	0.56578318	AR
R-HSA-194315	Signaling by Rho GTPases	0.557504007	0.557504007	AR
R-HSA-199991	Membrane trafficking	0.535061539	0.535061539	EGFR

### Molecular docking

**Figure 10:**
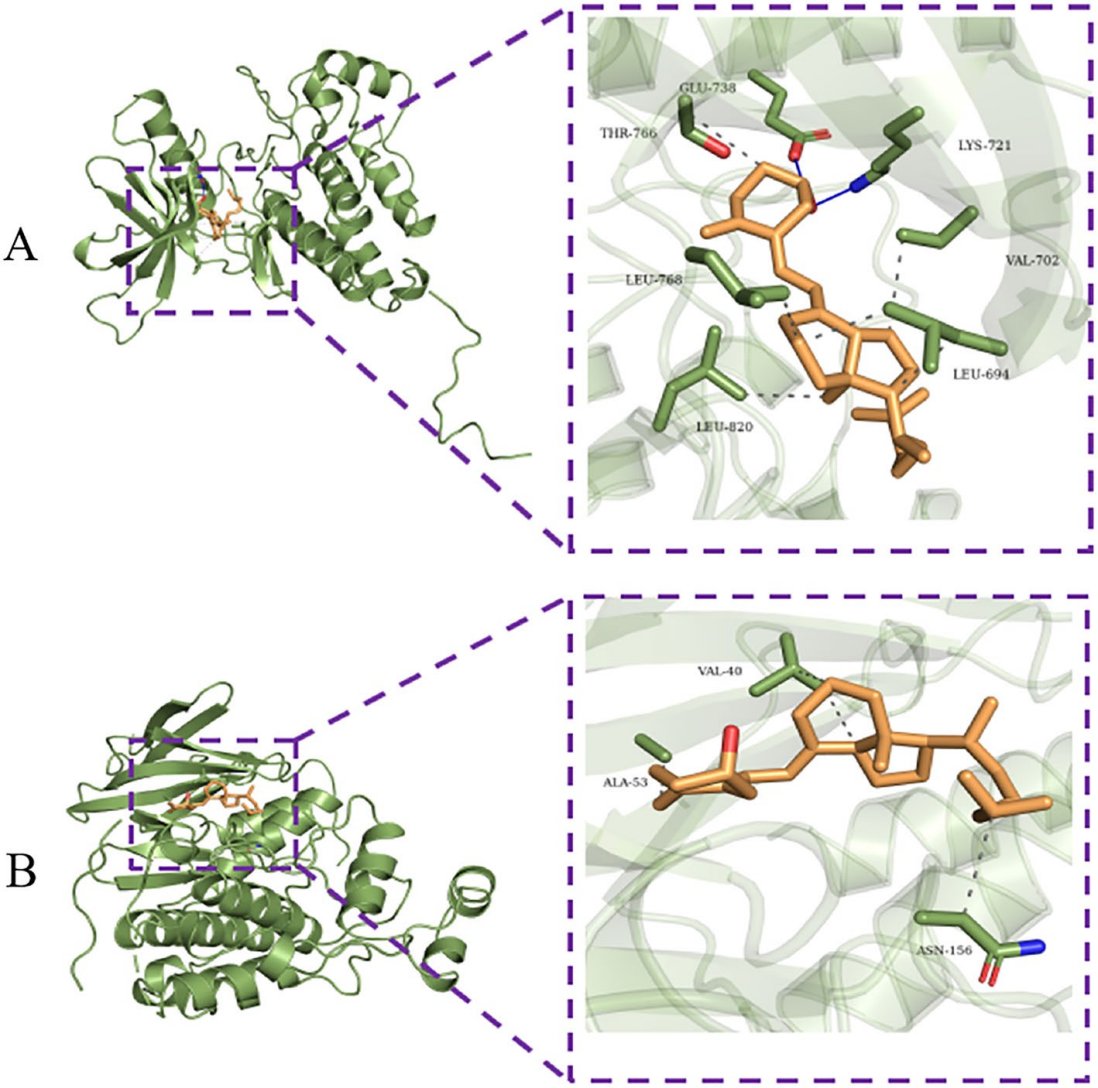
Docking pattern diagram of VitD with EGFR (**A**) and MAPK8 (**B**) The molecular docking pattern plots for VitD with EGFR (**A**) and MAPK8 (**B**), respectively.

Molecular docking technology is a virtual screening technology of molecular interaction based on computer-aided design, which plays an important role in studying the interaction between drug active ingredients and targets^32^. When the binding energy is < 0 kJ mol, the small molecule ligand can spontaneously bind to the protein receptor. If the binding energy is <  − 5.0 kJ mol or lower, it indicates that the two have the better binding ability^31^. Therefore, to further validate the results from the analyses of network pharmacology and bioinformatics, the molecular docking between the 2 critical common targets (EGFR, MAPK8) and VitD was performed. Through docking simulations, 2 pairs of docking results were yielded (Supplementary Table S20–21). Their binding energies were all <  − 5 kJ mol, indicating all of them can bind very well (Fig. 10). This molecular docking result indicates that the physical crosstalk between COVID-19 and osteoporosis may be realized by the interaction of VitD with the core targets (EGFR and MARK8) in the ERBB and MAPK signaling pathways.

## Discussion

COVID-19 is a serious infectious disease that spreads globally and threatens the health and lives of people worldwide since the end of 2019. Much evidence implicate a close association of VitD insufficiency to the pandemic and clinical manifestations of the COVID-19. Indeed, osteoporosis patients were found to be more susceptible to SARS-COV-2 infection and osteoporosis manifestations became worse after suffering from COVID-19 while some COVID-19 patients developed osteoporosis as a complication^33^. Therefore, VitD insufficiency may be a risk factor for both osteoporosis and COVID-19. VitD has been suggested as a potential adjuvant or alternative medicine for both osteoporosis and COVID-19^12^. It is currently unknown, however, whether and how VitD insufficiency links osteoporosis with COVID-19.

In this study, we applied network pharmacology and bioinformatics approaches and identified 2660 COVID-19 targets, 5093 osteoporosis targets, and 243 vitamin D targets. In the Venny diagram, we found that COVID-19 shared 855 targets with osteoporosis, implicating COVID-19 and osteoporosis may have shared mechanisms of pathogenesis. We also found that COVID-19, osteoporosis, and VitD shared 42 common targets. Therefore, VitD may mediate a crosstalk between COVID-19 and osteoporosis, which may explain the observations of that VitD insufficiency is closely associated with incidence and clinical manifestation of both COVID-19 and osteoporosis. We further constructed the DTD network of VitD-COVID-19-Osteoporosis based on the PPI network analysis, and 8 targets among the 42 common targets were initially screened as possible important core targets for VitD in the regulation of both COVID-19 and osteoporosis. The GO, KEGG, WikiPathways and Rectome pathway enrichment analyses on the 8 core targets further revealed the detailed mechanisms for VitD regulation of both COVID-19 and osteoporosis. GO analysis revealed that the biological process (BP) regulatory role of VitD mainly acted through cellular response to reactive oxygen species (ROS) and estrogen stimulation. Further screening of the signaling pathway databases, we found that ErbB and MAPK signaling pathway were co-expressed in KEGG and WikiPathways, while the activation pathway of AP-1 transcription factor family in KEGG endocrine resistance pathway is expressed in the immune system of Rectome. These shared pathways also shared common targets, namely ERBB2, EGFR and MAPK8. Among them, EGFR and MAPK8 targets are expressed in the immune system, innate immune system, cytokine signaling in the immune system, GPCR signaling, vesicle-mediated transport and other signaling pathways in the Rectome. Therefore, VitD may exert its regulatory effects mainly through ERBB2, EGFR and MAPK8 mediated activation of the ErbB and MAPK signaling pathways to control the cytokine storm process in COVID-19 patients and to ameliorate pulmonary fibrosis, and activation of osteoimmune mechanisms to regulate the structural integrity of bone. These targets and pathways are relevant for the formation of multiple immune defense mechanisms in COVID-19 and osteoporosis patients. Molecular docking simulations further validated the binding activity between VitD and its target proteins EGFR and MAPK8, reinforcing the importance of the aforementioned targets in the DTD network of VitD-COVID-19-Osteoporosis.

Previous studies have shown that acute SARS-CoV-2 infection often triggers cellular and humoral immune responses^34^, which can directly or indirectly damage the relevant cells in the respiratory tracts and leads to severe pulmonary fibrosis in COVID-19 patients^35^. The immune responses in patients with COVID-19 are manifested by marked lymphopenia and elevated serum pro-inflammatory cytokines, as well as a significant infiltration of mesenchymal lymphocytes in lung tissue and excessive activation of T cells in peripheral blood^36^. Plasma concentrations of IL-1β, IFN-γ, MCP-1 and IP-10 are elevated in COVID-19 patients, which may cause a Th1-type response. In addition, plasma concentrations of Th2 cytokines IL-4, IL-10 and IL-13 were also significantly upregulated in COVID-19 patients^37^. Thus, the immune response in COVID-19 patients is more inclined to Th1 and Th2 types. In clinically severe cases of COVID-19, an excessive immune response due to an overreaction of the immune system or because the immune system is too weak to control the replication of the virus, causes an inflammatory storm process, also called cytokine storm^38^, which aggravates the lung damage and causes death. It has been reported that VitD can delay the progression of pulmonary fibrosis^39^, and can be used as an adjuvant therapy for patients with pulmonary fibrosis^40^, although the underlying mechanisms are not clear.

The data from our study as described above suggested that VitD may block the immune response of the COVID-19 patients to reduce the pathological process of pulmonary inflammation and fibrosis. As shown in Figs. 7 and 8, we identified EGFR and ErbB signaling pathways as important targets and signaling pathway of pulmonary fibrosis in patients with COVID-19. In COVID-19 patients, pulmonary fibrosis occurs mainly due to the EGFR-mediated ErbB signaling pathway producing more pro-fibrotic than anti-fibrotic effects. SARS-CoV-2 infection rapidly activates inflammatory T cells and inflammatory monocytes/macrophages in the body, leading to the production of EGFR, AR and TGF-β, and IL-6^41^. These immune cells and inflammatory factors enter the lungs and exert immune damaging effects, leading to severe lung injury or even shock, which may be one of the causes of the SARS-CoV-2 infection induced inflammatory storm^42^. The ErbB family of receptor tyrosine kinases (RTKs) binds extracellular growth factor ligands to intracellular signaling pathways to regulate various biological responses, including proliferation, differentiation, cell motility, and survival. Ligand binding to four closely related members of this RTK family—EGFR (also known as ErbB-1 or HER1), ErbB-2 (HER2), ErbB-3 (HER3), and ErbB-4 (HER4)—forms homo- and heterodimers of the receptor, activates intrinsic kinase structures, resulting in acid phosphorylation of specific tyrosine residues (pY) in the cytoplasmic tail^43^. EGFR is characterized by autophosphorylation and phosphorylation of tyrosine residues in the cytoplasmic tail leads to activation of MAPK, JNK and Akt signaling pathways through the ErbB signaling pathway, which leads to inhibition of apoptosis, cell proliferation and migration, activation of inflammatory responses and increased mucus production, leading to lung injury^44^. EGFR signaling in fibrosis development has a bidirectional regulatory role. Studies have shown that TGF-β1 as a fibrosis inducer can effectively induce the expression of EGFR ligand AR. Silencing of AR using the EGFR-specific small molecule inhibitor gefitinib attenuated the fibrotic effects of TGF-β1^44^. In addition, mice overexpressing the EGFR ligand TGF-α also developed pulmonary fibrosis spontaneously, and similar effects existed for other EGFR ligands. These studies suggest that activation of EGFR signaling is pro-fibrotic^45^. On the other hand, a similar association with interstitial lung disease, a precursor of pulmonary fibrosis, has been observed in patients treated with the anti-EGFR monoclonal antibody panitumumab^46^. The EGFR-specific small molecule inhibitor gefitinib also exacerbated bleomycin-induced pulmonary fibrosis in mice. This suggests that activation of EGFR signaling has an anti-fibrotic effect^47^. Thus, the regulation of EGFR signaling on pulmonary fibrosis is bidirectional, and whether it is promoted or inhibited may depen Some of the above evidence suggests that while EGFR regulates pulmonary fibrosis, it also has a role in promoting bone formationd on the different disease states.

A lack of VitD is linked to chronic inflammatory lung diseases and respiratory infections because it regulates the host's defense mechanism against infections^48,49^. Through the induction of CYP24A1, it was discovered that TNF-α/IL-1 reduced the expression of the vitamin D-mediated antimicrobial activity hCAP18/LL-37, indicating that chronic inflammation impairs protective responses induced by VitD. Sp1 and the EGFR-MAPK pathway, two transcription factor-specific proteins, may play a role in the mechanism^48,50^. Additionally, it has been discovered that low VitD levels may create an environment that is conducive to the growth of tumors with EGFR mutations^51^. Low VitD levels have also been linked, according to Dong-Yeon Shin et al., to an increase in EGFR-mutant lung cancer cases^52^. There is more knowledge about how VitD influences the EGFR locus gene, particularly in lung diseases, to control the proliferative activity of many different cancer cells^53^. These findings collectively imply that VitD modulates EGFR genes to control lung inflammation, which may have important ramifications for the management of lung infections in patients with COVID-19.

As shown in Fig. 8, we found that MAPK8 and MAPK signaling pathway are also involved in the pathological process of COVID-19^43^. The involvement of MAPK signaling pathway in COVID-19 is less studied, but it is also closely associated with the major pathological process of pulmonary fibrosis^54^. As a downstream pathway of the ErbB signaling pathway, MAPK signaling can be activated both by the EGFR-mediated ErbB signaling pathway^55^ and by factors such as IL-1 and TNF^56^. In mammals, four main subtype pathways of MAPK signaling pathway exist, and three subtype pathways, p38MAPK, ERK1/2, and JNK, are found to jointly regulate a variety of important cellular physiological and pathological processes such as cell growth, differentiation, stress adaptation to the environment, and inflammatory response. The p38MAPK signaling pathway is phosphorylated in hypoxic environments to regulate inflammatory responses by regulating transcription factor activity and cytokine synthesis^57^. In addition, the p38MAPK pathway interacts with inflammatory cytokines. Activated p38MAPK can promote the expression and release of various pro-inflammatory cytokines (IL-1β, TNF-α) and induce cytokine storm. Meanwhile, macrophages can be activated by inflammatory cytokines, which then activate the p38MAPK signaling pathway. Other inflammatory cells can also be activated by the p38MAPK signaling pathway, such as the inflammatory aggregation of neutrophils^58^. These results suggest that the p38MAPK signaling pathway can induce the activation of some inflammatory cells and factors, which eventually cause the generation of inflammatory storms in the lung, leading to lung injury. Studies have shown that patients with idiopathic pulmonary fibrosis have extensive angiogenesis. Angiogenesis is mainly due to a disruption of the balance between stimulating and inhibiting vascular growth factors^59^. High expression of vascular endothelial growth factor (VEGF) strongly activates ERK1/2, leading to morphological changes in the vasculature, and the growth of neovascularization in the lung will accelerate pulmonary fibrosis^60^. Finally, as an important branch of the MAPK pathway, the JNK signaling pathway can also lead to lung fibrosis^61^, and the main mechanisms are that activation of JNK in epithelial cells leads to epithelial-mesenchymal transition (EMT) and cell death, and activation of JNK in lung fibroblasts leads to myofibroblast phenotype^62^. Therefore, MAPK signaling pathway mediates inflammatory storm, lung angiogenesis, fibroblast to myofibroblast conversion and pulmonary fibrosis development in COVID-19 patients. Inhibition of the ErbB and MAPK signaling pathways by VitD reduces TGF-β signaling and inhibits EGFR ligand-dependent phosphorylation and attenuates the cytokine storm generated by the immune response to SARS-CoV-2 infection^63^.

We have determined that VitD regulates the EGFR gene to reduce lung infection in COVID-19 patients. We also discovered that VitD reduces lung inflammation in COVID-19 patients by helping to control the MAPK signaling pathway. Through the p38 MAPK signaling pathway, 1, 25-dihydroxyvitamin D3 has been shown by Haihua Yang et al. to cause neutrophil apoptosis in people with chronic obstructive pulmonary disease^64^. As a result, VitD supplementation has the ability to significantly reduce the inflammatory reaction brought on by granulocyte aggregation. By focusing on PSAT1 expression in vivo and in vitro, vitamin D3 regulates the MAPK pathway and reduces pulmonary fibrosis, as demonstrated by Wenxiang Zhu^65^. In addition, calcitriol as a derivative of vitamin D, which reduces the early pulmonary inflammatory response and epithelial-mesenchymal transition caused by bleomycin in mice, and it may also reduce the levels of tumor necrosis factor alpha and macrophage inflammatory protein-2 during acute lung injury brought on by lipopolysaccharide in mice^66,67^. In conclusion, VitD can improve the pulmonary fibrosis process in COVID-19 patients through multiple targets of EGFR and MAPK and other multiple pathways.

There exists an intrinsic connection between the skeletal system and the immune system, which is termed osteoimmunity, where cytokines and signaling pathways are the bridges between the two intrinsic connection^60^. Therefore, the series of immune responses occurring in COVID-19 patients would also affect the skeletal system through osteoimmune responses, thus inducing osteoporosis or aggravating the condition of osteoporosis patients. The key immune cells of osteoimmunity are T cells, B cells, dendritic cells, and bone marrow macrophages. The RANKL/RANK/OPG regulatory system is the main signaling pathway of the bone immune response. The main cytokines that favor bone formation are IL-1, IL-6, IL-17, and TNF, and the main cytokines that aggravate bone destruction are IL-4, IL-13, and INF-γ^68^. Our data analysis found that SARS-CoV-2 infection was able to activate T cells and inflammatory monocytes/macrophages, led to the production of EGFR, AR and TGF-β, IL-6, where the MAPK signaling pathway is also a downstream pathway belonging to the RANKL/RANK/OPG regulatory system^69^. EGFR not only regulates pulmonary fibrosis, but also regulates bone structural integrity^70^. It was found that EGFR inhibited the expression of osteoblast (OB) transcription factors Runx2 and Osterix, thereby suppressing osteoblast differentiation^71^. EGFR stimulates OB proliferation and inhibits their differentiation by inhibiting the IGF-1R/mTOR pathway through ERK1/2-dependent upregulation of IGFBP-3^72^. OB derived from multipotent bone marrow mesenchymal stem cells (MSC) plays an important role in bone structural integrity^70^, but MSCs have a short survival time and require constant replenishment^71^. Insufficient MSC leads to low bone formation activity, which increases the risk of osteoporosis^60^. Chandra et al. found that the activation of EGFR signaling increased the number of MSC and facilitated the promotion of OB formation^61^. The main mechanism of this process is that EGFR promotes the proliferation and survival of osteogenic progenitor cells by increasing the expression of early growth response factor 2 (EGR2). EGR2 promotes the proliferation and survival of OB by increasing the anti-apoptotic protein MCL1 and decreasing the apoptosis of OB^61^. Some of the above evidence suggests that while EGFR regulates pulmonary fibrosis, it also has a role in promoting bone formation. The MAPK signaling pathway is a downstream of the bone immune RANKL/RANK/OPG regulatory system and is critical in controlling OB differentiation and skeletogenesis^73^. The basic composition of the MAPK pathway is a three-tier kinase response pattern that includes MAPK kinase kinase (MAP kinase kinase kinase, MKKK), MAPK kinase kinase (MKK), and MAPK, which can be activated sequentially and together regulate a variety of important cellular physiological/pathological processes such as cell growth, differentiation, stress adaptation to the environment, and inflammatory responses. And p38MAPK is primarily involved in OB differentiation, skeletogenesis, and skeletogenesis through a series of kinase reactions in OB differentiation, Osteoclast (OC) formation and apoptosis^64^. Studies have shown that p38MAPK is an important positive regulator of OB function and bone formation in vivo^74^. In various stages of osteoblast differentiation, p38 MAPK is crucial. By increasing the activity or expression of genes that code for transcription factors specific to osteoblasts, p38MAPK-mediated phosphorylation aids in the promotion of the osteogenic process^75^. The p38MAPK signaling pathway also enhances the osteogenic differentiation of mesenchymal cells and promotes the expression of osteoblast markers like ALP, OC, and collagen^76^. ERK in the p38MAPK transduction pathway plays an important role in OB proliferation, adhesion, extension, migration and integration. ERK1/2 positively regulates OB differentiation and inhibits chondrocyte differentiation^77^. ERK also affects RANKL to cause OC activation, leading to enhanced osteolysis^78^. Finally, JNK signaling is also involved in OB differentiation, OC formation and apoptosis^79^. Multiple factors stimulate intracellular signaling aggregation in the MAPK pathway, which affects OB and OC proliferation, differentiation, and apoptosis^78^. It has been shown that the synergistic presence of VitD with p38MAPK contributes to skeletal muscle growth and regeneration^75,76^. Therefore, our data support the lotion that VitD may improve osteoporosis by binding to MAPK signaling pathway mediated osteoimmunity.

In summary, in this study we identified and characterized 42 common targets of VitD on both COVID-19 and osteoporosis and 8 core targets in the DTD network of VitD-COVID-19-osteoporosis. These VitD targets involved in the ErbB and MAPK signaling pathways are critical for fibrotic diseases such as COVID-19 and ossification due to bidirectional regulatory role of this pathway in pro-fibrotic/anti-fibrotic disorder and bone formation/bone distraction respectively^80,81^. These findings provided novel mechanistic insights into the functional roles and molecular network of VitD in both COVID-19 and osteoporosis. VitD may be used as a marker of poor prognosis or a possible risk factor for both COVID-19 and osteoporosis and supplementation of VitD may have beneficial effects for prevention and treatment of these devastating diseases.

## Supplementary Information

Supplementary Figures.

Supplementary Legends.

Supplementary Tables.

## Data Availability

Raw data has been made available. All data generated or analysed during this study are included in this published article and its supplementary information files.

## Abbreviations

COVID-19: Coronavirus disease 2019
SARS-COV-2: Severe acute respiratory syndrome coronavirus-2
IFR: Infection fatality ratio
WHO: World Health Organization
VitD: Vitamin D
BP: Biological process
CC: Cellular components
MF: Molecular function
ROS: Reactive oxygen species
EMT: Epithelial–mesenchymal transition
OB: Mosteoblast
MSiC: Mesenchymal stem cells
OC: Osteoclast
