# Supplementary figures and images for: Shared mechanisms and crosstalk of COVID-19 and osteoporosis via vitamin D
Source: Sci Rep. 2022 Oct 28;12:18147. doi: 10.1038/s41598-022-23143-7 (PMC9614744; doi:10.1038/s41598-022-23143-7)

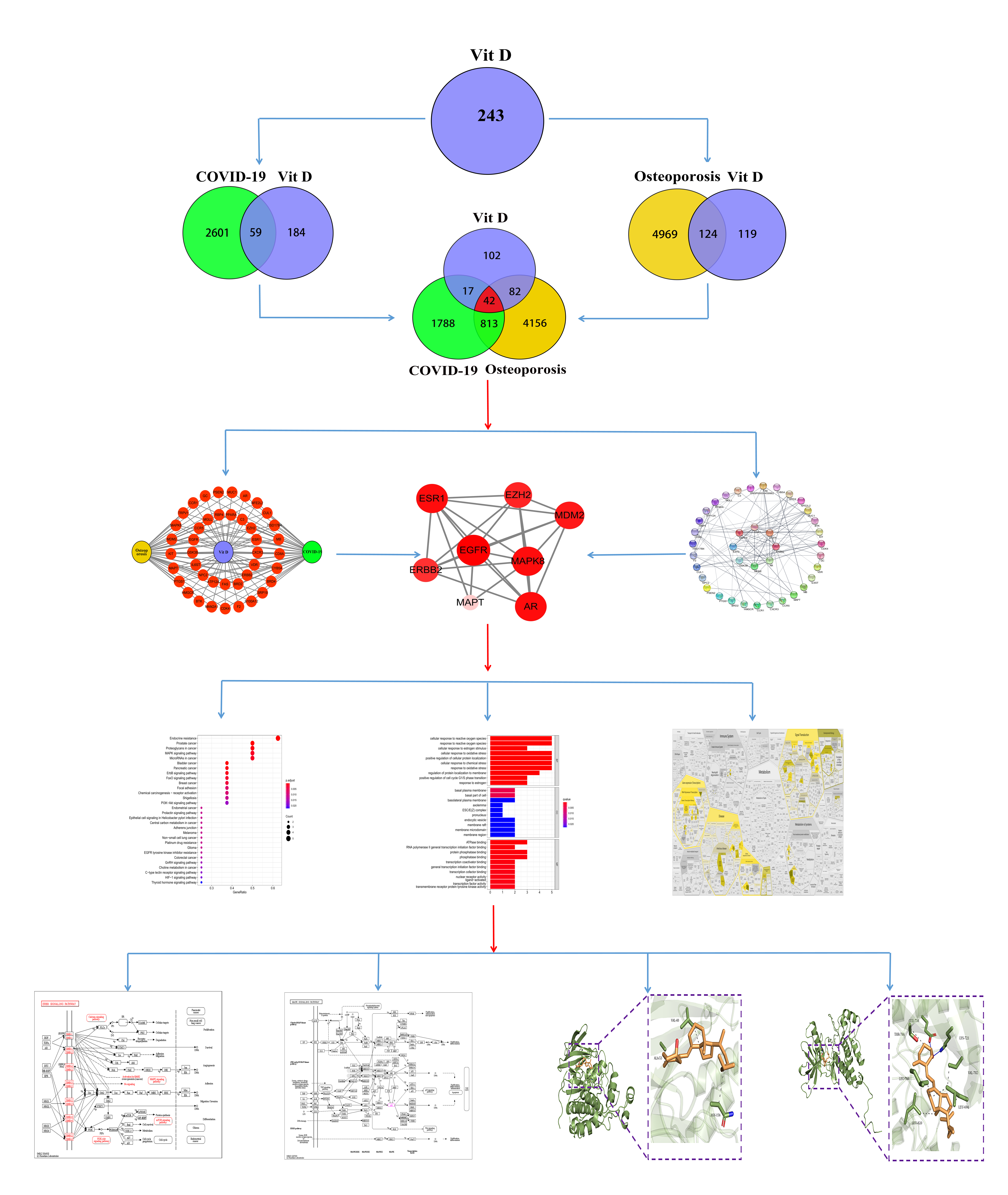

Supplement: Supplementary file 1 — Supplementary Figures. [file 41598_2022_23143_MOESM1_ESM.tif]
