# Supplementary material for: Shared mechanisms and crosstalk of COVID-19 and osteoporosis via vitamin D
Source: Sci Rep. 2022 Oct 28;12:18147. doi: 10.1038/s41598-022-23143-7 (PMC9614744; doi:10.1038/s41598-022-23143-7)
Supplement: Supplementary file 2 — Supplementary Legends. [file 41598_2022_23143_MOESM2_ESM.doc]

Table S1: This table shows the targets of action of vitamin D in the Drugbank database.

Table S2: This table shows the targets of vitamin D in the PharmMapper database.

Table S3: This table presents the targets of action of vitamin D in the pubchem database.

Table S4: This table shows the vitamin D targets in the SwissTargetPrediction database.

Table S5: This table shows the targets of osteoporosis in the DisGenet database.

Table S6: This table presents the targets of osteoporosis in the GeneCards database.

Table S7: This table presents the targets of osteoporosis in the NCBI database.

Table S8: This table shows the COVID-19 targets in the GeneCards database.

Table S9: This table shows the targets of COVID-19 in the NCBI database.

Table S10: This table shows the targets of COVID-19 in the TTD database.

Table S11: This table shows the combined action targets of vitamin D in the Drugbank, PharmMapper, pubchem, SwissTargetPrediction databases.

Table S12: This table shows the combined effect of osteoporosis targets in DisGenet, GeneCards, NCBI databases.

Table S13: This table shows the combined targets of COVID-19 in the GeneCards, NCBI, TTD databases.

Table S14: This table shows the intersection target data of the respective combined targets of vitamin D, osteoporosis, and new coronary pneumonia.

Table S15: This table presents the core targets for topological heterogeneity analysis of vitamin D, osteoporosis, and COVID-19 intersection target data.

Table S16: This table presents GO enrichment analysis based on core targets.

Table S17: This table presents KEGG signaling pathway analysis based on core targets.

Table S18: This table presents WikiPathway analysis based on core targets.

Table S19: This table presents Rectome assays based on core targets.

Table S20: This table presents EGFR and vitamin D molecular docking data.

Table S21: This table presents the data for molecular docking of MAPK and vitamin D.
